# Thinking points for effective batch correction on biomedical data

**DOI:** 10.1093/bib/bbae515

**Published:** 2024-10-13

**Authors:** Harvard Wai Hann Hui, Weijia Kong, Wilson Wen Bin Goh

**Affiliations:** Lee Kong Chian School of Medicine, Nanyang Technological University, 59 Nanyang Drive, Singapore 636921, Singapore; Lee Kong Chian School of Medicine, Nanyang Technological University, 59 Nanyang Drive, Singapore 636921, Singapore; School of Biological Sciences, Nanyang Technological University, 60 Nanyang Drive, Singapore 637551, Singapore; Lee Kong Chian School of Medicine, Nanyang Technological University, 59 Nanyang Drive, Singapore 636921, Singapore; School of Biological Sciences, Nanyang Technological University, 60 Nanyang Drive, Singapore 637551, Singapore; Center for Biomedical Informatics, Nanyang Technological University, 59 Nanyang Dr, Singapore 636921, Singapore; Center of AI in Medicine, Nanyang Technological University, 59 Nanyang Dr, Singapore 636921, Singapore; Division of Neurology, Department of Brain Sciences, Faculty of Medicine, Imperial College London, Burlington Danes, The Hammersmith Hospital, Du Cane Road, London W12 0NN, United Kingdom

**Keywords:** analysis, batch effects, biomedical informatics, data science, statistics

## Abstract

Batch effects introduce significant variability into high-dimensional data, complicating accurate analysis and leading to potentially misleading conclusions if not adequately addressed. Despite technological and algorithmic advancements in biomedical research, effectively managing batch effects remains a complex challenge requiring comprehensive considerations. This paper underscores the necessity of a flexible and holistic approach for selecting batch effect correction algorithms (BECAs), advocating for proper BECA evaluations and consideration of artificial intelligence–based strategies. We also discuss key challenges in batch effect correction, including the importance of uncovering hidden batch factors and understanding the impact of design imbalance, missing values, and aggressive correction. Our aim is to provide researchers with a robust framework for effective batch effects management and enhancing the reliability of high-dimensional data analyses.

## Teaser

Author summary: Batch effects introduce heterogeneity into data and hinder accurate analysis. Despite advancements in biomedical technologies and analysis algorithms, addressing batch effects remains a significant challenge due to their complex nature, which requires diverse considerations. To facilitate better handling of batch effects, we offer a comprehensive set of thinking points designed to help researchers ensure more robust analysis.

## Introduction

### What are batch effects?

Batch effects are systematic sources of heterogeneity that arise from factors other than the condition(s) of interest being studied. These factors include technical bias introduced by the use of different machines, instruments, environmental conditions, or handling personnel [1–3].

### Why are batch effects so important?

Batch effects introduce additional variability into data, which can significantly impact the interpretation of results and potentially lead to false associations [4, 5]. In biomedical settings, this may lead to misunderstandings about disease progression and origins [1]. To give one example: in a (retracted) study aiming to develop personalized treatment for ovarian cancer patients, gene expression signatures were falsely identified due to batch effects that were not corrected for [6]. Batch effects can also have profound influence on advanced predictive modeling applications, such as inaccurate identification of drug targets or wrong diagnosis/prognoses [7].

Batch effects are pervasive and important in any domain where instrumentation and high dimensional data are important. Fields like food sciences [8], environmental monitoring [9], engineering [10], and even education and social sciences [11] can contain batch effects in their data. Although batch effects may arise and be handled differently across various areas, their implications on data are fundamentally similar: they introduce skewed variations unrelated to the actual subject of study. If not properly addressed, this can lead to misleading conclusions.

The recent advent of artificial intelligence (AI) and machine learning (ML) has rendered batch effects even more important [3]. The quality of any AI/ML classifier is ultimately dependent on its input quality, which means good data preprocessing is important. It is known that incorrect handling of batch effects leads toward degradation of AI/ML performance [12–14]. However, while training data may be processed such that batch effects are minimized within itself, trained AI/ML classifiers are meant to perform tasks on new ‘unseen’ data that represents a different batch. Hence, new test data ‘induces’ a batch effect the moment it is presented to the trained classifier, thus requiring extra consideration.

### Understanding and correcting batch effects in biological data

**Figure 1:**
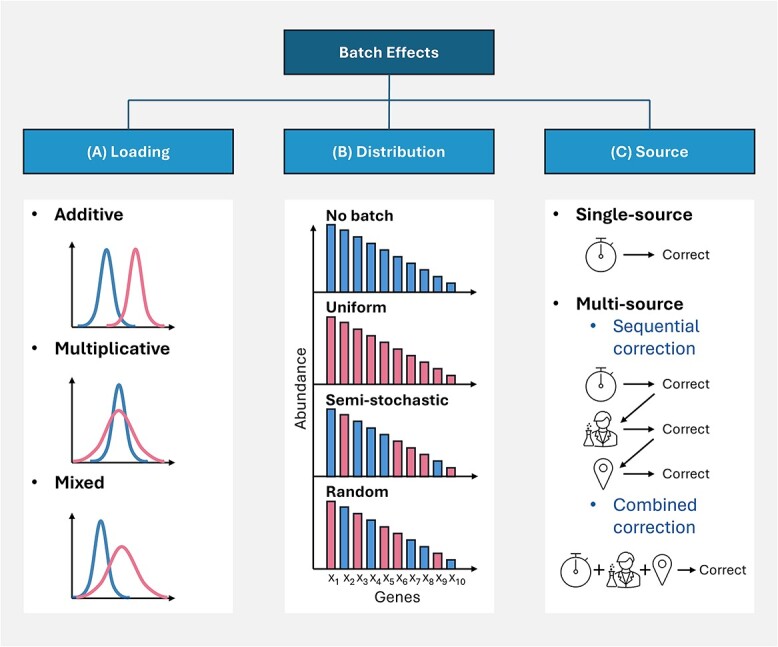
(A) Batch effects loading assumptions. Additive and multiplicative loadings indicate shifts in location and scale, respectively. Mixed loadings suggest both additive and multiplicative loadings. (B) Batch effect distributions using genes as an example. Uniform distributions indicate that all genes are affected by batch effects. Semi-stochastic distributions suggest that a subset of genes is more prone to incurring batch effects. Random distributions indicate that genes incur batch effects by pure chance. (C) Correction of single and multiple sources of batch effects. Multi-source batch effects require consideration on whether the batch effect sources should be corrected sequentially or collectively.

Batch effects encompass various technical biases that can arise during data generation, processing, and handling. Their diverse nature makes characterization challenging. To effectively address them, we must assume that batch effects fit certain theoretical assumptions, enabling us to develop appropriate correction strategies. We categorize these assumptions as loading, distribution, and source (Fig. 1).

The ‘loading’ assumption describes how a batch effect factor ‘loads’ information onto the original data. This loading can be additive, multiplicative, or a combination of both (mixed). (Fig. 1A). The loading assumption is used in ComBat, one of the best-known batch effect correction algorithms (BECAs) [15].

The batch loading may not be uniformly distributed across all features (e.g. genes, proteins). Moreover, some features may not carry any batch-related information. The sporadic nature of how batch effects influence individual features comes under the ‘distribution’ assumption. Like how missing values (MVs) are categorized [16–18], batch distributions can also be described as uniform, semi-stochastic, and random (Fig. 1B). In uniform batch effects, each feature is equally impacted by the batch factor. Random batch effects imply that each feature takes on the batch loading purely by chance. Semi-stochastic batch effects suggest that certain features are more likely to be influenced by batch effects than others, albeit seemingly randomly. This could be attributed to platform-specific issues or inherent properties of the features, such as their signal intensity or magnitude.

The final assumption is the batch effect ‘source’. Within a dataset, there may be more than one source of batch effects (Fig. 1C). These batch effects may influence each other but can also impact other factors [19]. When attempting to correct for batch effects, researchers may address only the most substantial batch effect (e.g. via regression analysis of the top principal component [20]) or deal only with those batch factors that are known to the experimenter. When dealing with multiple sources of batch effects, researchers must decide whether to address them sequentially or collectively.

### What is the gap?

Given the complexity and heterogeneity of batch effects, researchers should adopt a broader and more flexible approach to batch effects tailored to their research needs, rather than adhere rigidly to prescribed protocols. In this paper, we offer points for consideration in BECA evaluation, obstacles in batch effect correction, and on AI-based approaches.

### Batch effect correction algorithm evaluation

#### Consider the entire workflow—prioritize batch correction methods that are compatible with your entire data processing workflow, not just what is popular

Data processing workflows typically consist of a series of sequential steps, beginning with raw data acquisition and progressing through data matrix generation, normalization, MV imputation (MVI), batch effect correction, feature selection, and functional analysis [21]. Each preceding step influences the subsequent ones. Therefore, the choice of algorithm for each step should be compatible with the entire workflow, rather than just the immediately preceding or following steps.

In bulk gene expression analyses where the source of variation is known, commonly used BECAs include ComBat [15] and the removeBatchEffect() function in the limma R package [22]. Where the variation is unknown, Remove Unwanted Variation (RUV) [23, 24] or Surrogate Variable Analysis (SVA) [25] can be used. However, simply adopting these options by default is problematic as the BECA does not work in isolation but is influenced by other options taken in the workflow.

Our own recent work, OpDEA (https://github.com/PennHui2016/OpDEA) [26], showed that workflows are sensitive even to small changes. The overall compatibility of a BECA with the other workflow steps is important. Thus, it is crucial to check the assumptions of the BECA and ensure they are compatible with the rest of the workflow. Additionally, studying the interactions between BECAs and other workflow algorithms is beneficial. This approach will help create more effective workflows that synergize with the batch correction process.

One way is to use a method such as Select Batch-Correction Method (SelectBCM) [27]. SelectBCM applies a variety of BECAs on the user-input data before ranking the BECAs based on multiple evaluation metrics. While this speeds up the BECA selection process, we advise against relying on the top ranked BECA. Since the selection is based on a sum of ranks, it may be possible for a BECA to perform poorly in one evaluation metric but be redeemed by other metrics. Furthermore, using ranks may be unreliable if the variations in evaluation metrics between methods are small. For example, assuming two BECAs have very similar silhouette scores but have large differences in entropy—the difference in silhouette scores may not be very meaningful, unlike that of the entropy metric. Yet, with SelectBCM, the magnitudes of the differences are obscured, and are evaluated similarly. Therefore, while SelectBCM can be a convenient and effective tool, users should be aware of how to interpret its outputs. A quick check on the raw evaluation measurements between the top ranked BECAs can enhance the decision-making process when using methods like SelectBCM.

#### Use downstream sensitivity analysis to assess outcomes

**Figure 2:**
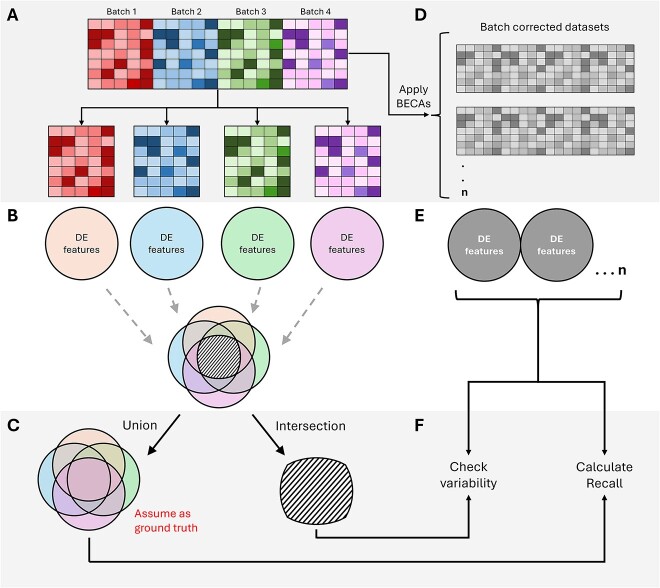
Performing sensitivity analysis to assess batch effect correction outcomes. (A) The dataset is separated into its individual batches. (B) Differential expression analysis is conducted on each batch to identify their differentially expressed (DE) features. These features are then aggregated. (C) The aggregated features are used to obtain two groups: the union, and the intersection. (D) Using the original data, apply the various BECAs that were identified for assessment and collect the corrected datasets. (E) Perform DE analysis to obtain the DE features from each of the corrected datasets. (F) Check the stability of each BECA by assessing the variability of the DE features obtained in (E) with the intersection of the DE features obtained in (C). The union of DE features obtained in (C) can serve as the ground truth to calculate the recall of each of the corrected datasets.

The authors of SelectBCM conceived the highly variable genes (HVG) union metric to assess the influence of BECAs on biological heterogeneity [27]. We found this concept useful and propose that a similar technique may be used to assess the reproducibility of downstream outcomes with different BECAs. Instead of HVG, we can look at the pool of differential features and compare both the union and the intersect of batches for a more stringent sensitivity analysis. Similar to SelectBCM, considering a variety of BECAs can be useful to get a sense of how findings could fluctuate if we used another algorithm (Fig. 2).

To identify a reliable method, we can start by comparing different batches and pinpointing differences in their outputs. Here, it is important that batches are comparable, as batches from vastly different sources (e.g. old and new technologies) may be too different to be integrated well in the first place. If comparable, we can first split the data into its individual batches (Fig. 2A), then perform differential expression analysis (DEA) on each batch to obtain their differentially expressed (DE) features and subsequently combining all unique features (Fig. 2B). The unique features will be stored by their union, as well as the intersect between batches, to serve as reference sets for later use (Fig. 2C). Next, we apply a variety of BECAs on the original data (Fig. 2D), before conducting DEA on each corrected dataset to obtain the respective DE features (Fig. 2E). With the union of DE features from the individual batches and those of each corrected dataset, we can calculate recall (correct identifications) and false positive rates (incorrect identifications) for each BECA, revealing the best performer (Fig. 2F). Additionally, DE features found in all batches (the intersect) can act as a quality check, where missing features after correction suggest underlying data issues potentially caused by the BECA itself. Finally, repeating this analysis across various datasets strengthens our understanding of the algorithms’ consistency.

#### Do not blindly trust visualization and batch metrics

Numerous methods for assessing batch effects are available, spanning from visualization aids to quantitative metrics. These evaluations offer insight into the extent of batch effects present in the data and may also indicate the performance of a BECA.

**Figure 3:**
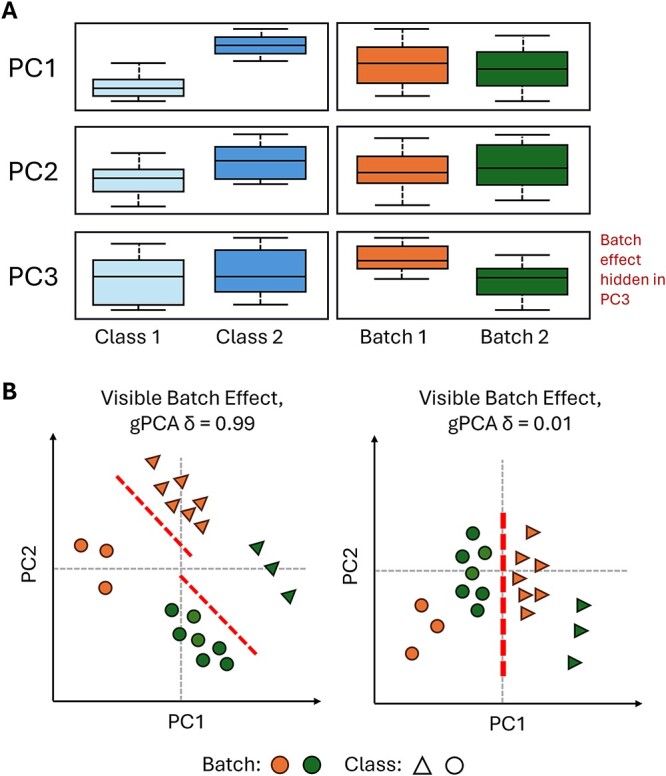
(A) Example of PCA visualization limitations. Plotting the PC1 and PC2 scatterplots will give the illusion of no batch effects. However, the batch effect may be hidden in PC3. (B) Illustration of how batch effect metrics can be unreliable. Dashed lines indicate the means of each batch. In both PCA plots, batch effects are clearly visible. However, the gPCA delta metric suggests that only the left PCA plot contains batch effects, simply because the batch means are the same in the right PCA plot.

A simple way to visualize batch effects in data is to build sample boxplots and compare the interquartile ranges [1]. However, this is unsuitable when the batch effects are complex or subtle. Another common approach is to reduce the dataset using a dimensionality reduction technique known as principal component analysis (PCA) [28]. PCA projects the data onto orthogonal vectors with the aim of identifying principal components (PCs) that preserve the variance of the data. Typically, the first two PCs from the PCA are visualized on a 2D-scatterplot, where samples are colored by batch. However, this only works if the batch effect is correlated with the first two PCs (which does not work for subtle batch effects corresponding to lower PCs). It is also highly inefficient to manually screen every PC scatterplot to uncover the batch correlated PCs. Instead, we may perform a quick statistical analysis on the PCs to identify the PCs that are associated with batch effects (Fig. 3A) [28, 29].

As PCA only captures linear relationships between features, data with non-linear relationships (e.g. single-cell omics) would not be suitable inputs. Two other dimensionality reduction methods, t-distributed stochastic neighbor embedding (t-SNE) [30] and uniform manifold approximation and project [31], are effective visualization tools for non-linear relationships in high-dimensional data through the capture of local data structures. The downside to these methods is that the global data structures are lost, and their outcomes are not interpretable nor useful for statistical analysis. Thus, these two tools are only useful for visualization purposes but should not be over-interpreted.

Besides visualization aids, various batch effect metrics have been proposed. Some are suitable for general usage, while others cater to specific characteristics of the data. For example, principal variance component analysis [32] and guided PCA (gPCA) [33] are two popular methods that use PCA to deduce batch effects in any PCA-appropriate data, while the silhouette width is a common metric used to evaluate clustering outcomes. These metrics can be useful but have limitations and are not always reliable (Fig. 3B). This was demonstrated in the simulation below where the experimental design was imbalanced, and the gPCA metric suggested that there were strong batch effects even though none had been added. A visual inspection of the PCA scatterplot, however, might contradict the metric and show that no batch effects are present.

### Tackling obstacles in batch effect correction

#### Explore unreported or unexpected sources of batch effects

Not all sources of batch effects are anticipated beforehand. While some are identified through experimental design, others, like the experimenter, reagent lots, or weather conditions, may be less apparent but still capable of introducing notable batch effects if overlooked [34]. Unfortunately, unreported batch sources are difficult to discern.

Singular value decomposition methods, such as SVA and RUV, protect important signals while removing other sources of variation and may seem capable of dealing with unreported batch sources. However, SVA is prone to over-correcting (removal of biological signal), especially when the biological signal is correlated to the latent batch effect [19]. Over-correction can be attenuated if we supply appropriate factor information to SVA to be protected. However, this entails extensive characterization and evaluation.

Yi et al. (2018) proposed a distribution-independent data-adaptive shrinkage and clustering (DASC) approach, which can identify subtle batch effects in omics data as covariates for downstream analysis [35]. Another related approach is pseudoreplicates of pseudosamples (PRPS) [36], which enables use of RUV-III even when batch factors are unknown. PRPS works by establishing expression–dependent biological populations in the data and sources of undesired variation, using these to create pseudosamples with biological signals and undesired variations that are approximately homogeneous. Pseudoreplicates are determined when pseudosamples are regarded to be from the same biological group. The PRPS are then used to calculate expression differences, which RUV-III can then use in conjunction with negative control genes to gauge batch effects in the data and subsequently eliminate them. Therefore, RUV-III with PRPS can enhance batch effect correction when unaccounted technical variations are introduced into the data.

**Figure 4:**
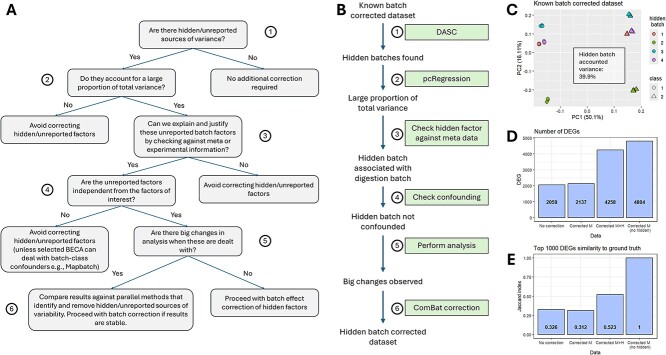
(A) Decision tree for dealing with hidden batch effect sources. (B) Workflow example using the hidden batch effect correction decision tree. (C) DASC correctly detected simulated hidden batches in the dataset. Principal component regression indicates that the hidden batch effect accounts for 39.9% of total variance. (D) Using a *t*-test, we compared the number of DEGs from the dataset without any correction (no correction), correction of the main batch effect (corrected M), correction of both main and hidden batch effect (corrected M + H), and correction of the main batch effect without hidden batch effects simulated [corrected M (no hidden)]. The corrected M (no hidden) dataset was taken as the ground truth. (E) Similarity of the top 1000 DEGs from the four datasets to the ground truth dataset. Since the ground truth dataset is compared to itself, a Jaccard index of 1 was obtained.

In Fig. 4A, we propose a decision tree for dealing with hidden sources of batch effects. When unreported batch sources are subtle, it may be better to avoid correcting them, lest we incur artifacts. However, the unreported sources become a concern when they account for a large proportion of total variance in the data. If they are confounded with the biological factor of interest, we should correct them using BECAs that can handle batch-class confounders, such as MapBatch [37]. Otherwise, we apply standard BECAs on the unreported factors, but not without comparing analysis outcomes before and after correction to assess the impact of the unreported factors. If a large difference is observed in the analysis, then the correction of the unreported factors should be justified by examining the metadata. It may also be useful to compare multiple methods of batch effect correction and unreported batch effect detection. This helps to establish the validity of the decision on whether to correct the unreported batch sources.

As a case study, we used the MultiPro mass spectrometry (MS)-based proteomics dataset containing two cell lines (HCC1806 versus HS578T) and two technical batches [38]. Four hidden batches were simulated into the dataset by adding random noise of mean 0 and standard deviation 0.5 into each hidden batch. Figure 4B describes the workflow we used when following the proposed decision tree. Using DASC, we correctly identified the four hidden batches from parameter tuning, and principal component regression [39] determined that the hidden batches accounted for a high proportion of total variance (Fig. 4C). Differential analysis revealed that the dataset corrected for both the main and hidden batch effect performed the most similarly to the original dataset corrected for the main batch effect without hidden batches simulated (Fig. 4D and E). Certainly, our case study presents a simplistic example of correcting hidden batch effects that may not necessarily reflect complex batch effects in nature. However, it illustrates the important fact that addressing hidden batch effects, when possible, can indeed lead to better outcomes for analysis.

#### Be mindful of batch and class effect imbalances

In a well-balanced experimental setup, most BECAs are expected to yield comparable performance [5]. However, batch-class imbalances are challenging for many BECAs, as they may mistake the class differences as batch effects (over-correction). On the other hand, BECAs might inadvertently mask the class effect while residual batch effects remain, potentially escaping detection depending on the sensitivity of the batch effect identification tool, leading to miscorrection. Some BECAs have built-in functionalities that attempt to handle such imbalanced scenarios by estimating and preserving class differences. Although this may address the imbalance issue, it has the undesirable side-effect of inflating *P* values [13]. We recommend comparing the correction outcomes with and without class covariates (supplied to the BECA) to determine the extent to which the results are due to the inclusion of the covariate.

While batch imbalance can directly confuse BECAs, another way batch imbalance can impact outcomes is through the process of data normalization. Normalization is a key part of the data pre-processing pipeline to ensure comparability between samples. When batches are imbalanced, normalization may cause an issue termed ‘test-set bias’, which relates to how samples are adjusted based on other samples in the dataset [40]. We can intuitively appreciate how the normalized outcome of the sample can differ when its surrounding samples are different. For example, in an imbalanced scenario where most samples in the data belong to a certain batch, cross-batch normalization will likely skew the normalized values toward the majority batch. Because of this, normalization should not be conducted mindlessly, especially when groups are imbalanced.

**Figure 5:**
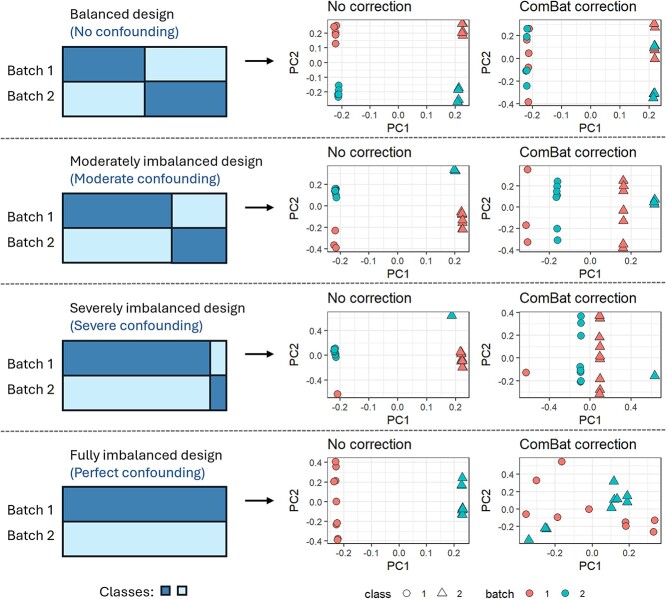
Four levels of experimental design confounding. ComBat correction when confounding is present causes miscorrection, where the biological signal is interfered. Confounding worsens in severity as classes become increasingly over-represented in a particular batch.

Imbalance issues also impact data models. Across four experimental designs—no confounding, moderate confounding, severe confounding, and perfect confounding (Fig. 5), Soneson et al. (2014) showed that when batch effects are present in a balanced design, correction before classification is likely to improve cross-validation classifier performance [14]. However, they reported that even a moderate imbalance in the design leads to high misclassification rates, which are unsalvageable even when a BECA is applied. It is worth noting that the authors in this study used ComBat with class covariates included (except in perfectly confounded scenarios), reinforcing the notion that this option does not deal with imbalance with the same effectiveness as in a balanced design. Hence, when the study design is imbalanced, we should not blindly trust the performance of the classifier during cross-validation, especially when only internal data is used. External data should also be included to serve as a true test for the classifier.

#### Handling missing values and batch effects

Missing values (MVs) are pervasive in biomedical data [17]. MVs affects data analysis by creating statistical uncertainty and, like batch effects, are complex to handle. MVs are usually corrected by MVI methods and are typically handled separately from batch effects. However, these issues are confounded [41].

**Figure 6:**
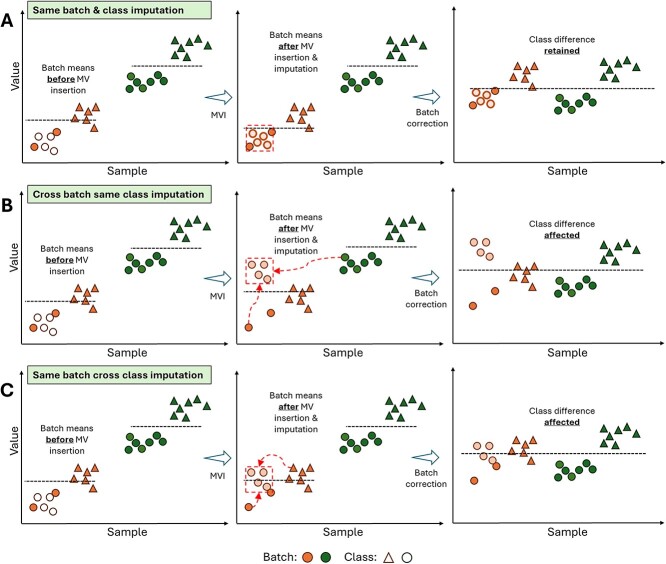
Illustration of how MVI potentially induces batch-related artifacts. White points represent values that were converted to MVs. Light orange points represent imputed MVs. (A) The ideal imputation scenario where the imputation is derived using information from the same batch and class. (B) Imputation relies on information from the same class of a different batch. The class becomes confounded with the batch, reducing effect size, and hindering batch effect correction. (C) Imputation relies on information from the same batch of a different class. In this case, because the batch effect is stronger than the class effect, a same batch imputation produces better results than in (B). However, the shift in batch means causes poor batch effect correction and leaves remnant batch effects.

Typically, MVI is performed before batch effect correction. However, this causes problems if batch information is not considered carefully during MVI (Fig. 6). We showed that imputation without regard for batch information increases intra-sample variance while also increasing false positives and false negatives [41]. The desired outcome for MVI is therefore achieved when batch information is contained within the same batch (Fig. 6A). However, this is not always possible, especially when using methods such as K-nearest neighbors where the number of observations in one batch may be less than the chosen hyperparameter K. In this case, the next most similar samples would either come from a different batch of the same class (Fig. 6B) or from the same batch of a different class (Fig. 6C). In either case, the batch effect correction is hindered, and class differences are affected.

Hence, we agree with recent best practice guides such as proBatch [1], which advocate against MVI before batch effect correction. However, performing batch effect correction before MVI is also challenging.

One way to achieve batch effect correction without MVI is to use HarmonizR [42]. HarmonizR corrects batch effects by sectioning data into smaller segments that are relatively free of MVs. It then corrects these segments independently using either ComBat or limma, before stitching back the data to form a batch-corrected matrix with MVs still intact. This makes HarmonizR an effective method for processing batch effects when the analysis steps do not require complete data. However, while HarmonizR can correct batch effects in MV-laden data, it does not address issues related to data completeness. According to the authors, imputing the data after applying HarmonizR is not recommended, as batch confounders may be reintroduced. Therefore, when MVI is required, the HarmonizR approach may be inappropriate and standard workflows may be required.

Given the prevalence of MVs and batch effect tangling in single-cell studies, recent developments have aimed at creating tools that can handle MVs, correct batch effects, and denoise the data all together. A single-cell RNA-seq (scRNA-seq) propensity score matching method (scPSM) that also relies on mutual nearest neighbors pairs matching was proposed to accomplish the three goals, simultaneously correcting batch effects and imputing MVs in the data to avoid potential confounders [43]. In the emerging field of single-cell proteomics, the recently developed single-cell PROTeomics EmbeddINg (scPROTEIN) similarly performs the three goals through a single framework based on cell graph construction and contrastive learning. However, while scPSM performs imputation, scPROTEIN compensates for missingness indirectly by ‘borrowing’ information from similar cells through the shared cell graph [44]. Methods like scPSM and scPROTEIN that are specifically designed to synchronize the handling of MVs and batch effects warrant greater exposure, as these innovative approaches have shown great benefit in retaining the true biological signal in the data, improving subsequent downstream analysis.

Moreover, as alluded in the previous paragraph, batch effects are not always about the loadings or modulation of observable values. The non-random distribution of MVs can also be a manifestation of batch effects. Batch effect associated missing values (BEAMs) are when the distribution of MVs is batch-specific [16].

BEAMs are extreme batch-associated MVs such that in one batch, there is a pronouncedly high degree of missingness resulting in the imputation process becoming biased toward only a few batch-specific samples. We first encountered BEAMs when we found that given the same sample, technical replicates across machines generated missingness rates of 10%, 30% and 50%, respectively (data obtained from the Clinical Proteomic Tumor Analysis Consortium) [45].

In such situations, even if we accounted for batch effects during MVI, we could do little about the BEAMs. A global correction is not appropriate, as the imputation is then driven primarily by the batch with least missingness. The severity of this complication worsens in situations where features are only identified in certain batches or in a single batch, meaning that any errors from those batches is propagated into other batches and may be mistaken as the correct signal. Situations of such missingness were described by Brenes et al. (2019), where overall missingness of the data remained similar when additional batches were added due to the increasing number of features detected in only a single batch [46]. BEAMs are a challenging scenario, but it is important as it can emerge during the process of data integration. Although this problem is becoming increasingly noticed, there is limited knowledge for dealing with BEAMs. We think this is an important area that could be addressed by the research community.

#### Be careful not to obscure heterogeneity in your data

**Figure 7:**
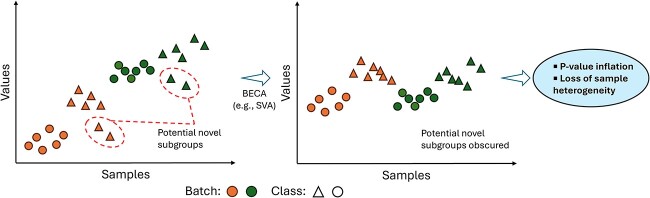
Demonstration of how aggressive batch effect correction obscures biological heterogeneity. Potentially novel sample subgroups with personalized expression signatures are removed along with the batch effect after BECA application. Increased homogeneity inadvertently inflates *P* values.

BECAs that emphasize on supplied class covariates (e.g. ComBat with class covariates) are known to aggressively correct batch effects while preserving class differences. However, this process also removes biological heterogeneity and thus personalized expression signatures that are unrelated to the class covariate, along with the batch effect (Fig. 7). As a result, accurate identification of novel subtypes from batch-corrected data is challenging when using standard algorithms designed to remove batch effects for class comparison analyses. Furthermore, obscuring biological heterogeneity has been shown to increase class differences and inflate *P* values, leading to unreliable feature selection outcomes [47, 48]. These BECAs are also impractical in genomics-based clinical trials, where the biological groups are unknown a priori and thus cannot be supplied for preservation.

A classic example of aggressive correction was demonstrated by Zindler et al. (2020), where they showed that false discovery rates and Bonferroni-corrected false positives rose to concerningly high levels following batch effect correction of microarray data with ComBat [48]. This was true even with balanced designs, in contrast to previous studies which suggested that aggressive correction was a problem that required attention only in imbalanced designs [13]. While increasing the number of samples may reduce the impact of aggressive correction, it cannot entirely avoid the problem. In addition, they showed that when more batches exist in the data, the number of false positive cases may grow exponentially.

SVA was another BECA subject to criticism over concerns about its tendency to exaggerate group differences, as its function depended on known class factors in the data and estimating and removing variances not associated with the classes. This prompted the development of permuted-SVA (pSVA) [47], which supposedly solved the class-inflation issues that SVA had by accepting batch factors into its algorithm rather than class factors. In this sense, the algorithm is blind to the biological covariates, similar to ComBat without including the covariate option. Unfortunately, while pSVA did perform better than ComBat with class covariates in terms of preserving biological heterogeneity, a comparison with its non-covariate counterpart was not conducted. However, it would be logical to assume that BECAs that are blind to class covariates are less likely to aggressively correct.

On a similar note, batch-effects-correction-with-unknown-subtypes (BUS) [49] tries to correct batch effects in data with the aim of preserving unknown biological subgroups. It uses a location and scale adjustment model similar to ComBat [50], with an additional model-based clustering component to guess the biological subgroup information for each sample. Like pSVA, BUS requires only the batch factors to be known, but not the class factors. However, the drawback is that the number of subgroups in the data must be supplied, which means that users must be aware of the hidden subgroups in the first place. The upside of BUS is that it can detect unevenness in batch subgroups, where subgroups are present only in certain batches of data.

The main limitation of methods like ComBat, pSVA and BUS is that they require known technical factors, but these are not always available. A potential work-around is to use DASC to detect hidden batch factors which can then be supplied to BECAs. Alternatively, we may also consider deep learning (DL) methods like batch effect signature correction (BESC) [51], which, like SVA, does not require batch factors as an input but is devoid of the issue regarding sample heterogeneity. BESC uses a group of predetermined samples as a reference to identify batch effect signatures (BES) to estimate batch effects in new samples through a linear model. Although not implicitly stated, the ability of BESC to correct data at the level of individual samples implies that it can be suitable for imbalanced data as well. Currently, BESC is limited to data obtained from two types of microarray data platforms, which severely limits its potential. However, its core concept is certainly worthy of investigation in other domains, especially its potential to correct imbalanced data without incurring false positives.

Various BECAs used in single-cell studies were developed in response to challenges with over-correction [52, 53]. In other words, bio-conservation can be achieved in these studies using a wide variety of BECAs. Many such BECAs are based on AI techniques, which we discuss in greater detail in the following section.

#### Leverage artificial intelligence to enhance the effectiveness of batch correction

AI approaches, encompassing tasks like classification and latent representations, are increasingly prominent in batch effect correction endeavors due to their strong performances and ability to overcome certain obstacles in batch effect correction. DL-based BECAs are particularly prominent in scRNA-seq studies, where datasets contain extensive observations across batches. These DL methods excel in addressing complex, nonlinear problems, thus improving batch effect correction outcomes beyond what traditional BECAs like ComBat or SVA can achieve, such as discerning rare subgroups within batches. As dataset sizes expand, DL methods become crucial, as simplistic assumptions of traditional BECAs struggle to cope with increased complexity. However, DL methods are unlikely to entirely supplant traditional BECAs, as they necessitate substantial data volumes, making their application challenging in fields with limited sample sizes due to concerns of overfitting. Additionally, traditional BECAs maintain utility due to their interpretability and computational efficiency. While not universally applicable, DL methods are highly relevant in the era of big data. Thus, alongside addressing technical challenges encountered by traditional BECAs, exploration of advanced AI's capacity to understand and utilize sample characteristics is essential.

In the preceding section, we briefly discussed the use of a DL-method, BESC, which learns from a set of data before applying batch effect correction on other samples [51]. While BESC works by training on a pre-built library, many DL-based BECAs in scRNA-seq make use of autoencoders. These autoencoders are heavily enabled by the substantial number of observations in each batch, allowing the algorithm to learn complex functions about the underlying structure of the data. Examples of such methods include single-cell variational inference (scVI) [54], Batch Effect ReMoval Using Deep Autoencoders (BERMUDA) [55], BERMAD [53], DESC [56], and Mapbatch [37].

ScVI acquires a probabilistic representation of scRNA-seq data by employing conditional variational autoencoders, considering factors such as batch factors, biological signals, and random noise [54]. This representation enables the embedding of cells into a latent space devoid of batch effects, thereby reflecting genuine biological disparities. An added advantage to scVI is that by sampling from the underlying distribution, it can also facilitate differential expression analysis. Single-cell ANnotation using Variational Inference (scANVI) is a semi-supervised extension to scVI, using known labels in the input to improve cell annotation accuracy and therefore bio-conservation [52]. When these labels are available, scANVI has been shown to outperform various other BECAs [57].

While scVI depends on cell similarities and thus does not maximize the clustering of distinct cell populations, BERMUDA applies transfer-learning to project the data into a low-dimensional feature space, which enables clustering of similar cell types with homogenous batch-mixing [55]. As previously mentioned, over-correction is often an issue in single-cell studies—something that both scVI and BERMUDA may struggle with. To address this, Zhan et al. (2024) [53] proposed BERMAD, a multi-layer adaptation autoencoder that also processes each batch separately to capture and preserve batch-specific biological signals, reducing both over-correction and under-correction concerns. DESC similarly learns a low-dimensional representation of the data and focuses on optimizing a clustering objective function through iterative learning [56]. Unlike scVI, BERMUDA, and BERMAD, DESC does not require batch factor input and is also more computationally efficient which gives it an edge in practical usage especially when faced with large scRNA-seq data. However, if cell types are unique to certain batches, lapses in the clustering are likely to occur in these methods which may lead to incorrect batch effect correction.

Mapbatch consists of an ensemble of autoencoders that each learn the data structure of different cell types from single samples [37]. Unlike the aforementioned BECAs, its main priority is to achieve conservative batch effect correction while preserving the biological signal. In principle, it is similar to BESC, though it involves a more sophisticated ML algorithm to learn from the input data itself. The strength of Mapbatch lies in the ensemble of autoencoders trained on individual samples, which when multiple batches are involved in training, allows it to retain biological signals from cell populations that are unique to certain batches.

The strength of AI methods for batch effect correction is widely recognized, as evidenced by the plethora of recent advancements [58–63]. Some methods, such as CLEAR, have diverged from using autoencoders [63]. CLEAR leverages self-supervised contrastive learning to achieve strong clustering and batch effect correction. Unlike the aforementioned methods, which generally rely on unsupervised autoencoders, the self-supervised nature of CLEAR allows users to guide the training process, overcoming a key challenge that most unsupervised methods face: explainability of the constructed low-dimensional embeddings. This contributes to strong performances that can also be interpreted with biological relevance. In addition, CLEAR is highly scalable to large datasets, making it a flexible yet powerful tool for batch effect correction in scRNA-seq data.

Besides DL methods, other ML methods are also relevant in batch effect correction. Harmony, for example, corrects batch effects by iteratively clustering similar cells from different batches in a reduced feature space, applying a correction factor to minimize batch differences within cell clusters until convergence [64]. Another method, linked inference of genomic experimental relationships (LIGER), uses integrative non-negative matrix factorization to capture dataset-specific factor loadings, which are then supplied to enhance the clustering of similar cells, thereby removing the batch effect [65]. Therefore, while DL is common in AI methods for batch effect correction, innovative alternatives continue to see development and remain relevant in the current space.

#### Batch effect correction algorithm selection chart

**Figure 8:**
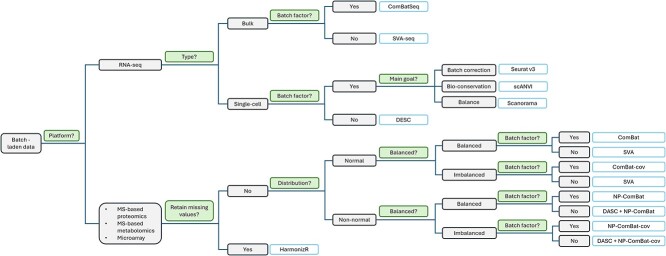
Decision chart for selecting a BECA given a batch-laden dataset. Note that BECA selection in scRNA-seq is complex and can depend on many other considerations not shown in this chart.

Due to the overwhelming list of BECAs available, selecting an optimal method can sometimes be difficult. To help readers quickly decide on a BECA, we provide a non-exhaustive list of various BECAs and their required inputs and outputs (Table 1), and a decision chart that covers some key factors in BECA selection (Fig. 8). First, we identify the platform from which the data is obtained. If it is RNA-seq, we must determine whether bulk sequencing or single-cell sequencing is involved. For bulk sequencing, the BECA can be determined by the availability of the batch factor, where we select ComBatSeq [15] if available and SVA-seq [66] if not. For scRNA-seq, DESC can be used if the batch factor is unknown, but the main goal must be clearly defined when the batch factor is available. If strong batch correction is desired, Seurat v3 [67] can be applied. For a more conservative correction, and if some cell annotations are known, scANVI can be useful. Otherwise, Scanorama [68] has been shown to result in a more balanced correction that achieves both batch correction and bio-conservation [57]. However, we would like to emphasize that BECA selection in scRNA-seq remains a difficult task due to the sheer number of considerations involved, such as computational complexity and output format etc. While our recommendations for single-cell BECAs are based on well-known benchmarking studies [57, 69], users should remain aware of other considerations that can affect BECA performance.

**Table 1:** A collection of BECAs available for biomedical data

BECA	Platform	Input	Output	Source/package
ComBat/NP-ComBat [50]	MS-based proteomics, MS-based metabolomics, microarray	1) Normalized-expression matrix 2) Class factors 3) Batch factors	Batch corrected expression matrix	SVA R package
ReComBat [70]	MS-based proteomics, MS-based metabolomics, microarray	1) Normalized-expression matrix 2) Class factors 3) Batch factors	Batch corrected expression matrix	reComBat python package https://github.com/BorgwardtLab/reComBat
HarmonizR [42]	MS-based proteomics, MS-based metabolomics, microarray	1) Normalized-expression matrix 2) Batch factors	Batch corrected expression matrix	HarmonizR R package
SVA [25]	MS-based proteomics, MS-based metabolomics, microarray	1) Normalized-expression matrix 2) Class factors	Expression matrix with non-class related variation removed	SVA R package
BMC [71]	MS-based proteomics, MS-based metabolomics, microarray	1) Normalized-expression matrix 2) Batch factors	Batch corrected expression matrix	bapred R package
BUS [49]	MS-based proteomics, MS-based metabolomics, microarray	1) Normalized-expression matrix 2) Batch factors	1) Batch corrected expression matrix 2) Estimated subtypes 3) Batch effect distribution parameters	BUScorrect R package
RUV [24]	MS-based proteomics, MS-based metabolomics, microarray	1) Normalized-expression matrix 2) Class factors	Expression matrix with non-class related variation removed	RUVnormalize R package
M-ComBat [72]	MS-based proteomics, MS-based metabolomics, microarray	1) Normalized-expression matrix 2) Class factors 3) Batch factors	Batch corrected expression matrix	Github R package https://github.com/SteinCK/M-ComBat
fSVA [73]	MS-based proteomics, MS-based metabolomics, microarray	1) Normalized-expression matrix 2) Class factors	Expression matrix with non-class related variation removed	SVA R package
pSVA [47]	MS-based proteomics, MS-based metabolomics, microarray	1) Normalized-expression matrix 2) Batch factors	Batch corrected expression matrix	SVA R package
FAbatch [71]	MS-based proteomics, MS-based metabolomics, microarray	1) Normalized-expression matrix 2) Class factors 3) Batch factors	Batch corrected expression matrix	bapred R package
Harman [20]	MS-based proteomics, MS-based metabolomics, microarray, bulk RNA-seq	1) Normalized-expression matrix 2) Class factors 3) Batch factors	Batch corrected expression matrix	Harman R package
Limma [22]	MS-based proteomics, MS-based metabolomics, microarray, bulk RNA-seq, scRNA-seq	1) Normalized-expression matrix 2) Batch factors	Batch corrected expression matrix	limma R package
RUV-seq [23]	Bulk RNA-seq	1) Normalized-expression matrix 2) Class factors	Expression matrix with non-class related variation removed	RUVSeq R package
ComBatSeq [15]	Bulk RNA-seq, scRNA-seq	1) Normalized-expression matrix 2) Class factors 3) Batch factors	Batch corrected expression matrix	SVA R package
SVA-seq [66]	Bulk RNA-seq, scRNA-seq	1) Normalized-expression matrix 2) Class factors	Batch corrected expression matrix	SVA R package
Harmony [64]	scRNA-seq	(Seurat object) 1) Normalized-expression matrix 2) Class factors 3) Batch factors	Batch corrected embeddings	Github R package https://github.com/satijalab/seurat
Seurat v3 canonical correlation analysis [67]	scRNA-seq	(Seurat object) 1) Normalized-expression matrix 2) Batch factors	Batch corrected expression matrix	Github R package https://github.com/satijalab/seurat
Seurat v3 reciprocal PCA [67]	scRNA-seq	(Seurat object) 1) Normalized-expression matrix 2) Batch factor	Batch corrected expression matrix	Github R package https://github.com/satijalab/seurat
scVI [54]	scRNA-seq	(AnnData object) 1) Raw expression matrix 2) Batch factors	Batch corrected embeddings	scvi-tools python package

(*Continued*)

**Table 1:** Continued

BECA	Platform	Input	Output	Source/package
scANVI [52]	scRNA-seq	(AnnData object) 1) Raw expression matrix 2) Class factors 3) Batch factors	Batch corrected embeddings	scvi-tools python package
LIGER [65]	scRNA-seq	(Seurat object) 1) Normalized expression matrix 2) Batch factors	Batch corrected embeddings	Github R package https://github.com/welch-lab/liger
Scanorama [68]	scRNA-seq	(AnnData object) 1) Normalized expression matrix 2) Batch factors	Batch corrected embeddings	Github python implementation https://github.com/brianhie/scanorama
Autoencoder-based Batch Correction (ABC) [74]	scRNA-seq	(AnnData object) 1) Normalized expression matrix 2) Class factors 3) Batch factors	Batch corrected expression matrix	Github python implementation https://github.com/reutd/ABC
Semi-supervised integration of single-cell transcriptomics data [75]	scRNA-seq	(Seurat object) 1) Normalized expression matrix 2) Class factors 3) Batch factors	Batch corrected embeddings	Github R package https://github.com/carmonalab/STACAS
BERMUDA [55]	scRNA-seq	1) Normalized expression matrix 2) Cluster pairs 3) Batch factors	Batch corrected expression matrix	Github python implementation https://github.com/txWang/BERMUDA
DESC [56]	scRNA-seq	(AnnData object) Normalized expression matrix	1) Cluster assignments 2) Cluster probabilities 3) Dimension-reduced data	Github python implementation https://github.com/eleozzr/desc
BERMAD [53]	scRNA-seq	1) Normalized expression matrix 2) Batch factors	Batch corrected expression matrix	Github R package https://github.com/ zhanglabNKU/BERMAD
Contrastive LEArning framework for single-cell RNA-sequencing (CLEAR) [63]	scRNA-seq	(AnnData object) Normalized expression matrix	1) Cluster assignments 2) Batch corrected embeddings	Github python implementation https://github.com/ml4bio/CLEAR
scPSM [43]	scRNA-seq	1) Normalized expression matrix 2) Batch factors 3) Marker genes	Batch corrected expression matrix	Github R package https://github.com/eleozzr/scPSM
scPROTEIN [44]	Single-cell proteomics	1) Normalized expression matrix 2) Peptide/Protein list	Batch corrected embeddings	Github python implementation https://github.com/TencentAILabHealthcare/scPROTEIN

For MS-based proteomics, MS-based metabolomics, and microarray derived data, we continue to split by four levels. Firstly, we decide whether to retain MVs in data or not. If we do, then HarmonizR is the most appropriate BECA. If not, we then look at the distribution, whether the design is balanced, and whether the batch factor is known. In general, when the distribution is not normal, we use non-parametric ComBat (NP-ComBat). When the design is imbalanced, we use a BECA that includes class covariates, such as ComBat with covariates (ComBat-cov) or SVA. If the batch factor is unknown, we use SVA if appropriate, and if not, we first identify hidden batch factors with DASC before using an appropriate version of ComBat.

## Conclusion

Batch effects are pervasive and significant sources of variability in high-dimensional data across various domains. They are complex and their presence can lead to misleading conclusions if not properly addressed. In this paper, we highlight the importance of adopting a more flexible and holistic approach to batch effect correction, emphasizing the need to consider the entire data processing workflow, potential obstacles, and the impact of AI-based approaches.

**Key Points**

- Batch effects are a major challenge in biological data analysis due to their complex nature.
- Selection of BECAs should depend on workflow compatibility, evaluation techniques, and sensitivity analysis outcomes.
- Hidden batch effects, design imbalance, MVs, and aggressive correction can impede correction performance.
- AI approaches can potentially enhance batch effect correction processes.

## Data Availability

The R code and dataset used to generate the case study analysis can be found at: https://github.com/HarvardHui/BatchCorrectionThinkingPoints.
